# Serological measures to assess the efficacy of malaria control programme on Ambae Island, Vanuatu

**DOI:** 10.1186/s13071-017-2139-z

**Published:** 2017-04-26

**Authors:** Zulkarnain Md Idris, Chim W. Chan, Mubasher Mohammed, Morris Kalkoa, George Taleo, Klara Junker, Bruno Arcà, Chris Drakeley, Akira Kaneko

**Affiliations:** 1 Island Malaria Group, Department of Microbiology, Tumor and Cell Biology, Karolinska Institutet, Stockholm, Sweden; 2 Department of Parasitology and Medical Entomology, Faculty of Medicine, Universiti Kebangsaan Malaysia Medical Centre, Kuala Lumpur, Malaysia; 3 Ministry of Health, Port Vila, Vanuatu; 4 Department of Immunology and Infection, London School of Hygiene & Tropical Medicine, London, UK; 5 Yeast and Fermentation, Carlsberg Research Laboratory, Copenhagen, Denmark; 6 Department of Public Health and Infectious Diseases, Parasitology Section, Sapienza University of Rome, Rome, Italy; 7 Department of Parasitology and Research Centre for Infectious Disease Sciences, Graduate School of Medicine, Osaka City University, Osaka, Japan; 8 Institute of Tropical Medicine, Nagasaki University, Nagasaki, Japan

**Keywords:** Malaria, Serology, Island, ITN, *Plasmodium falciparum*, *Plasmodium vivax*, *Anopheles*

## Abstract

**Background:**

Seroepidemiology can provide evidence for temporal changes in malaria transmission and is an important tool to evaluate the effectiveness of control interventions. During the early 2000s, Vanuatu experienced an acute increase in malaria incidence due to a lapse in funding for vector control. After the distribution of subsidised insecticide-treated nets (ITNs) resumed in 2003, malaria incidence decreased in the subsequent years. This study was conducted to find the serological evidence supporting the impact of ITN on exposure to *Anopheles* vector bites and parasite prevalence.

**Methods:**

On Ambae Island, blood samples were collected from 231 and 282 individuals in 2003 and 2007, respectively. Parasite prevalence was determined by microscopy. Antibodies to three *Plasmodium falciparum* (PfSE, PfMSP-1_19_, and PfAMA-1) and three *Plasmodium vivax* (PvSE, PvMSP-1_19_, and PvAMA-1) antigens, as well as the *Anopheles*-specific salivary antigen gSG6, were detected by ELISA. Age-specific seroprevalence was analysed using a reverse catalytic modelling approach to estimate seroconversion rates (SCRs).

**Results:**

Parasite rate decreased significantly (*P* < 0.001) from 19.0% in 2003 to 3.2% in 2007, with a shift from *P. falciparum* predominance to *P. falciparum*-*P. vivax* co-dominance. Significant (*P* < 0.001) decreases were observed in seroprevalence to all three *P. falciparum* antigens but only two of three *P. vivax* antigens (except PvAMA-1; *P* = 0.153), consistent with the more pronounced decrease in *P. falciparum* prevalence. Seroprevalence to gSG6 also decreased significantly (*P* < 0.001), suggesting that reduced exposure to vector bites was important to the decrease in parasite prevalence between 2003 and 2007. Analyses of age-specific seroprevalence showed a three-fold decrease in *P. falciparum* transmission, but the evidence for the decrease in *P. vivax* transmission was less clear.

**Conclusions:**

Serological markers pointed to the effectiveness of ITNs in reducing malaria prevalence on Ambae Island between 2003 and 2007. The recombinant gSG6 antigen originally developed to indicate exposure to the Afrotropical vector *An. gambiae* may be used in the Pacific to complement the traditional measure of entomological inoculation rate (EIR).

## Background

In recent years, analyses of antibody responses to one or more malaria-specific antigens have been widely used to assess transmission intensity [1–3], temporal and spatial reductions in transmission [2, 4], as well as to confirm elimination [5, 6]. In areas of low transmission, long-lasting antibody responses may be easier to detect than parasite prevalence in human populations or infected mosquitoes [2], making serological measures useful adjunct metrics to measure malaria transmission. Recently, serological evidence of exposure to malaria has demonstrated successful interventions in some low-transmission settings [7–9].

Located at the margin of malaria transmission, Vanuatu is an archipelago of more than 80 islands in the South Pacific. Malaria is endemic on most of the nation’s 68 inhabited islands [10]. The majority of infections are due to *Plasmodium falciparum* and *Plasmodium vivax*, with some rare cases of *Plasmodium malariae* [10, 11]. There are two seasons in Vanuatu: the dry and cool season from May to October, and the wet and hot season from November to April. Hence malaria incidence in Vanuatu shows seasonal fluctuations, which are more pronounced for *P. falciparum* than *P. vivax* [10, 12]. The only known malaria vector is *Anopheles farauti* (*s.s*.), a member of the *Anopheles punctulatus* complex [13].

Several large-scale control programmes have been implemented in Vanuatu over the past decades. In 1988, insecticide-treated nets (ITNs) were introduced; by 1992 ITN coverage had reached 27% of Vanuatu’s population [10]. The sharp decline in malaria incidence during the 1990s was explained by the equitable ITN distribution that targeted the most vulnerable populations i.e. children under five years, their mothers, and pregnant women [12]. An integrated malaria elimination programme was initiated on the southern-most island of Aneityum in 1991 [14]. By the mid-1990s, with a high degree of commitment from the local communities, malaria had been eliminated from Aneityum [14, 15]. An acute increase in malaria incidence was observed after shortages in funding for malaria control in the early 2000s, followed by a decline in incidence after the resumption of distribution of subsidised ITNs. Since 2009, significant external support has enabled the expansion of intervention measures including the distribution of free long-lasting insecticide treated-bed nets (LLINs) to cover more than 80% of the population, focal indoor residual spraying (IRS), the introduction of free rapid diagnostic test (RDT) and free artemisinin-based combination therapy (ACT) to all health facilities, and enhanced surveillance and rapid response to identified cases. Further decrease in malaria incidence since 2009 has put Vanuatu on course to achieve malaria elimination in the near future [16].

This study reports on the parasitological and sero-epidemiological results from samples collected on Ambae Island in 2003 and 2007. In addition to responses to antigens from *P. falciparum* and *P. vivax*, exposure to vector mosquito bites was also evaluated, with the aim to determine the effect of vector control on parasite prevalence. The three parasite antigens used in this study vary in immunogenicity. The *Plasmodium* crude schizont extract (SE) is multi-antigenic and has greater sensitivity to detect low residual transmission and changes in transmission intensity [7, 17]. The blood-stage antigen merozoite surface protein-1_19_ (MSP-1_19_) exhibits moderate immunogenicity and has been useful for estimating malaria transmission in populations across a gradient of transmission intensity [1]. The blood-stage apical membrane antigen-1 (AMA-1) is highly immunogenic and induces long-lived immune responses. In moderate transmission settings, seroconversion occurs more rapidly for AMA-1 than MSP-1_19_ [2, 7]. The *An. gambiae* salivary gland antigen (gSG6) showed strong immunogenicity among African populations in previous studies [18, 19]. The short-lived antibodies against gSG6 appear to correlate with changes in *Anopheles* abundance [20], which is useful for evaluating vector control strategies [21].

## Methods

### Study sites and sample collection

**Fig. 1:**
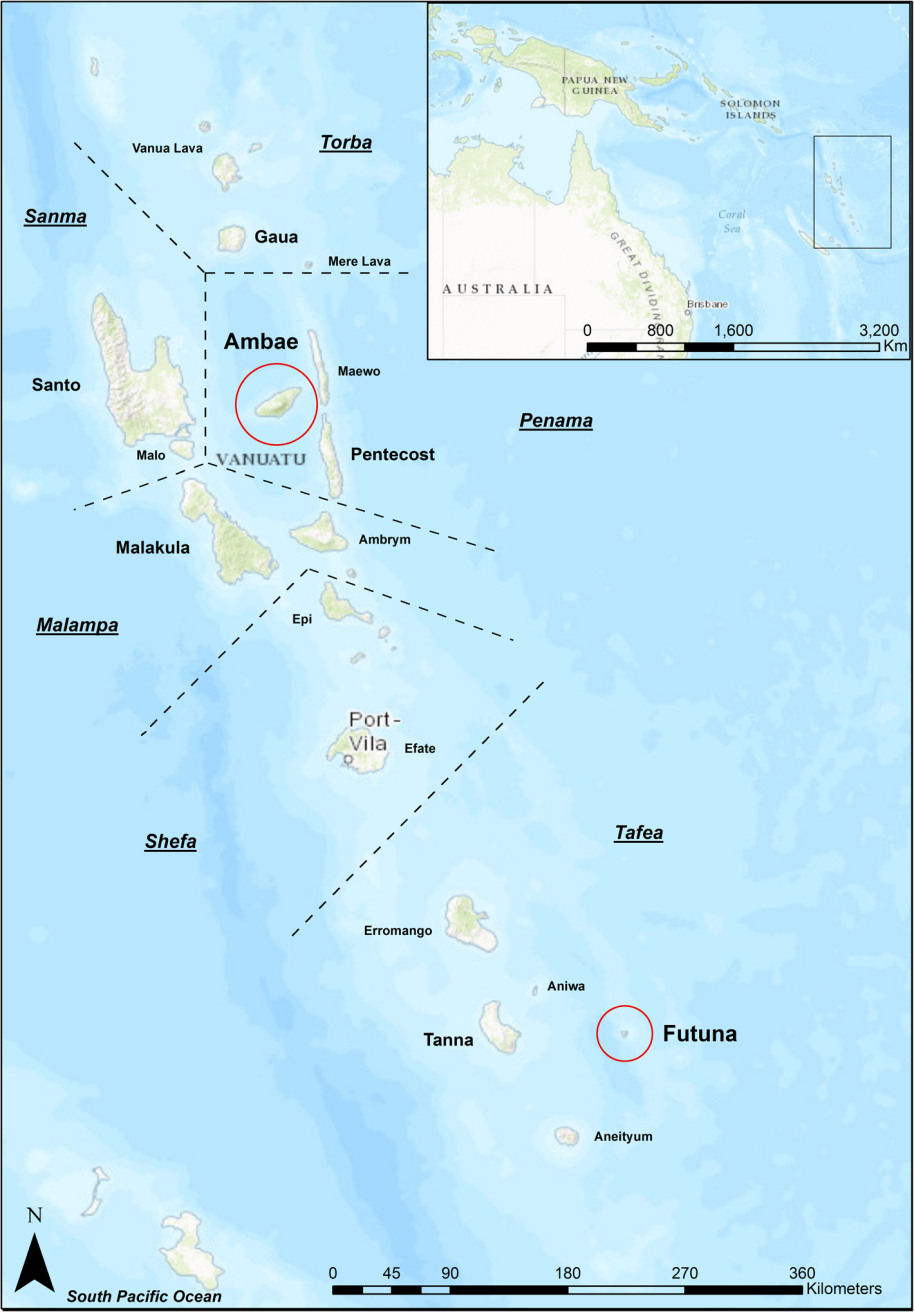
Locations of Ambae Island and Futuna Island in Vanuatu**.** The names of the six provinces in Vanuatu are underlined, and approximate provincial boundaries are indicated by dashed lines. Inset shows the location of Vanuatu. The map was created with ArcGIS software, version 10.4, http://www.esri.com/

Ambae Island (398 km^2^) is in Penama Province (Fig. 1) and has a population of 10,407 (Vanuatu National Census, 2009). Transmission of *P. falciparum*, *P. vivax* and *P. malariae* is ongoing, and the transmission intensity is characterised as meso-endemic [10, 22]. Samples were collected during cross-sectional malariometric surveys conducted in south Ambae in June 2003 (*n* = 231) and June 2007 (*n* = 282). Futuna Island (11 km^2^) is in Tafea Province (Fig. 1) and has a population of 535 (Vanuatu National Census, 2009). In contrast to Ambae, Futuna lies beyond the Buxton line, which defines the southeastern limit of anopheline mosquito breeding, thus has always been free of malaria [10, 14, 17, 23, 24]. Samples were collected in July 2011 (*n* = 392) and were included in the analyses of parasite prevalence and vector exposure only.

Informed consent was obtained from all participants. The consent procedure was witnessed by a third party (e.g. teacher, village chief), who also recorded the name of each participant as he/she enrolled in the survey. Gender and age were recorded for each participant. Axillary body temperature was determined using a digital thermometer (Terumo, New Jersey, US). Fever was defined as a temperature exceeding 37.5 °C. Spleen size was assessed in children aged 12 years and younger according to Hackett’s method by one investigator only (AK). Blood samples were obtained for microscopic examination of malaria infections. Two spots of blood (70 μl each) were collected on Whatman 31ET Chr filter paper (Whatman, Maidstone, UK). The blood spots were air-dried and stored in plastic bags at ambient temperature in the field and later at -20 °C in our laboratories until processing. This study was approved by the Ministry of Health in Vanuatu and by the Committee on the Ethics of Human Research of Karolinska Institutet in Sweden.

### Parasite prevalence by microscopy

Thin and thick blood smears were stained with 3% Giemsa solution for 30 min and examined under oil immersion (1,000× magnification) by experienced microscopists. Blood smears were defined as negative if no parasites were found after examining 100 high power microscopy fields. For all positive samples, malaria species were identified, and the presence of *P. falciparum* gametocytes was recorded.

### Assay of anti-malarial antibody responses

A blood spot (3 mm in diameter) was punched from each sample and antibodies were eluted in reconstitution buffer in 0.5 ml deep well plates (Corning Costar, PA, USA) as described previously [25]. The reconstituted blood spot solution, equivalent to a 1:200 dilution of serum, was stored at 4 °C until use.

All sera from Ambae were tested for IgG antibodies by indirect quantitative enzyme-linked immunosorbent assay (ELISA) to crude schizont extract (SE), the 19 kDa fragment of recombinant merozoite surface antigen-1 (MSP-1_19_), and recombinant apical membrane antigen-1 (AMA-1) for *P. falciparum* (PfSE, PfMSP-1_19_ [Wellcome strain], and PfAMA-1 [3D7 strain]) and *P. vivax* (PvSE, PvMSP-1_19_ [Belem strain], and PvAMA-1 [Sal-1 strain]) as described previously [25]. Crude SE of *P. falciparum* and *P. vivax* antigens were prepared as previously described [26, 27]. Briefly, NUNC-Immuno plates (Sigma-Aldrich, St. Louis, USA) were coated with 50 μl of coating buffer containing SE, AMA-1 and MSP-1_19_ at 0.5 μg/ml. The plates were washed in PBS with 0.05% Tween 20 (PBS/T) and blocked using 1% (w/v) skimmed milk (Sigma-Aldrich) in PBS/T for three hours. After washing, 50 μl of reconstituted serum were added in duplicate. The final antigen dilutions were 1:1000 for SE and MSP-1_19_, and 1:2000 for AMA-1. In addition, four blank wells and a five-fold dilution series of an African hyper-immune serum pool (*n* = 12) were added per plate. The plates were washed and 50 μl of horseradish peroxidase (HRP)-conjugated rabbit anti-human IgG antibody (DAKO, Glostrup, Denmark) was added at a dilution of 1:15,000 in PBS/T and incubated for 3 h. After further series of washes, antibody responses were detected after development with 100 μl of the substrate solution 3, 3', 5, 5'-Tetramethylbenzidine (TMB) (tebu-bio laboratories, Le Perrey-en-Yvelines, France) for 15 min. The reaction was stopped with 50 μl of 2 M H_2_SO_4_. The optical density was read using Multiskan Go ELISA reader (Thermo Fisher Scientific, Waltham MA, USA) at 450 nm.

All samples from Ambae and Futuna were tested for exposure to bites of *An. farauti* (*s.s*.) using the recombinant *An. gambiae* salivary gland gSG6 antigen [19]. ELISA was performed as described above for the parasite antigens, with the exception that NUNC-Immuno plates (Sigma-Aldrich) were coated with the recombinant antigen at 5 μg/ml in 50 μl of coating buffer (final antigen dilution of 1:100).

### Statistical methods

Data were double-entered and imported in STATA/SE version 13.1 (StataCorp, TX, USA). Optical density (OD) values were averaged and normalised against values from blank wells to adjust for background reactivity as previously described [25]. Seropositivity was determined by fitting a mixture model to normalised OD values assuming two Gaussian distributions, one for seronegative individuals and another for seropositive individuals [3]. The mean OD plus three standard deviations associated with the seronegative group was used as the cut-off value for seropositivity. A separate cut-off was generated for each antigen. Differences in proportions were tested using the Chi-squared test or the Fisher’s exact test. Antibody levels among populations were compared using the Mann-Whitney U test or the Kruskal-Wallis test with Dunn’s multiple comparison *post-hoc* tests. Pairwise correlations between ODs of different antibody responses were determined using the Spearman’s rank correlation. Seroprevalence was stratified into yearly age groups and then analysed using a reverse catalytic modelling approach under a binomial sampling assumption, as described elsewhere [1, 9, 28]. This provides an estimate of the mean annual rates of conversion to seropositive (seroconversion rate, SCR [λ]) and reversion to seronegative (seroreversion rate, SRR [ρ]), averaged over the age of the population. Infants under 1 year of age were excluded to remove any influence of maternally derived antibodies [1]. Logistic regression was used to identify factors associated with seropositivity to any parasite-specific antigens and gSG6. In the regression analysis, seropositivity to *Plasmodium* was defined as being positive for either or both species-specific antigens (MSP-1_19_ and/or AMA-1). Survey year, gender, age group, fever, and infection status were considered as explanatory variables in the univariate analyses. All variables with a *P*-value of ≤ 0.05 from the likelihood ratio test in the univariate analyses were included in the multivariate logistic regression model. The variables included in the final (adjusted) model was survey year and age group.

## Results

### Characteristics of the study population and parasite prevalence

In total, 231 and 282 people were sampled from Ambae in 2003 and 2007, respectively; 392 people were recruited from Futuna in 2011. Most the sampled populations were 20 years old and younger. On Ambae, the gender ratio and age distribution of the samples did not differ between 2003 and 2007 (Table 1). The prevalence of fever, enlarged spleen in children, as well as *Plasmodium* infections were significantly lower in 2007 than in 2003 (Table 1; Chi-square test or Fisher’s exact test: all *P* < 0.01). In both sampled years, most *Plasmodium* infections were found in children 10 years and younger (Fig. 3a). *Plasmodium malariae* was found in only two individuals in 2003, both of whom were co-infected with *P. falciparum*. The decrease in prevalence was more apparent for *P. falciparum* (-9.9%) than *P. vivax* (-5.1%), resulting in a shift in species composition from the predominance of *P. falciparum* in 2003 to the co-dominance of *P. falciparum* and *P. vivax* in 2007. The prevalence of *P. falciparum* gametocytes also decreased significantly (Fisher’s exact test: *P* < 0.001) between the sampled years (Table 1).

**Table 1:** Sample characteristics, clinical and parasitological results for all surveyed islands by year [%, (*n*)]

		Ambae			Futuna	
Characteristic	Category	2003	2007	*P-*value^a^	2011	*P-*value^b^
		(*n* = 231)	(*n* = 282)		(*n* = 392)	
Gender	Female	52.8 (122)	53.9 (152)	0.859	51.0 (200)	0.752
Median age (IQR^c^)		16 (7–27)	12 (6–26)	0.653	14 (8–25)	0.877
Age group in years	0–5	17.7 (41)	23.0 (62)	0.268	15.8 (62)	0.120
	6–10	22.5 (52)	20.2 (57)	0.588	18.9 (74)	0.552
	11–20	24.2 (56)	19.5 (55)	0.198	35.7 (140)	< 0.001
	> 20	35.5 (82)	38.3 (108)	0.522	29.6 (116)	0.051
Fever (> 37.5 °C)		8.2 (19)	0.0 (0)	< 0.001	8.2 (32)	< 0.001
Enlarged spleen (< 12 years old)		69.8 (74/106)	28.8 (23/80)	< 0.001	2.9 (2/70)	< 0.001
Parasite prevalence		19.0 (44)	3.2 (9)	< 0.001	0 (0)	< 0.001
Species-specific prevalence	*P. falciparum*	11.7 (27)	1.8 (5)	< 0.001	0 (0)	< 0.001
	*P. vivax*	6.5 (15)	1.4 (4)	0.004	0 (0)	< 0.001
	*P. malariae*	0.9 (2)	0.0 (0)	0.204	0 (0)	0.054
Gametocyte prevalence^d^	*P. falciparum*	7.8 (18)	0.7 (2)	< 0.001	0 (0)	< 0.001

^a^ Proportion or median level of each category between two surveys on Ambae Island (2003 and 2007)

^b^ Proportion or median level of each category among all three surveys (Ambae Island in 2003 and 2007 and Futuna Island in 2011)

^c^ IQR: interquartile range (25–75th percentile)

^d^ No *P. vivax* and *P. malariae* gametocytes were detected by microscopy

As expected, no malaria infection was detected on Futuna. The prevalence of enlarged spleen in children on Futuna was significantly lower (Fisher’s exact test: both *P* < 0.001) than that from Ambae in both 2003 and 2007. The prevalence of fever on Futuna was similar to that on Ambae in 2003 (Table 1). These fever cases were likely caused by other infections, but they were not explicitly investigated during our survey.

### Breadth and correlation of different antibody responses

**Fig. 2:**
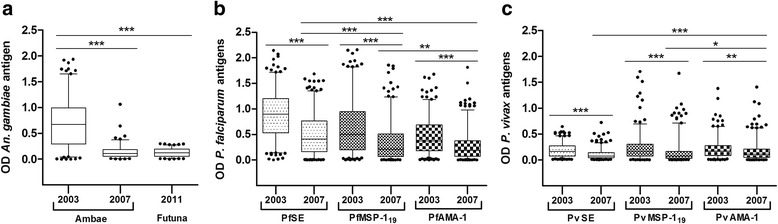
Species-specific antibody responses to *An. gambiae* salivary gland gSG6 antigen (**a**), *P. falciparum* antigens (**b**), and *P. vivax* antigens (**c**). The interquartile range (IQR, 25–75th percentile) is represented in a box plot with the median OD value shown as a line within the box.*P < 0.05; **P < 0.01; ***P < 0.001

Antibody levels as measured in optical densities are shown in Fig. 2. The median anti-gSG6 antibody level showed a significant (Mann-Whitney U-test: *Z* = 6.57, *P* < 0.001) decrease on Ambae between 2003 and 2007. No significant difference in gSG6 antibody levels was observed between Ambae in 2007 and Futuna, where *Anopheles* vectors are absent. Similar to the decrease in anti-gSG6 antibody levels, median antibody levels to all *P. falciparum* and *P. vivax* antigens also decreased significantly (Kruskal-Wallis H-test: all *P* < 0.01) on Ambae between the survey years. Significant (Kruskal-Wallis H-test: all *P* < 0.05) differences in median antibody levels were observed among parasite-specific antigens on Ambae in 2007. For *P. falciparum*, the anti-SE antibody level was significantly (Mann-Whitney U-test: both *P* < 0.01) higher than the anti-MSP-1_19_ and anti-AMA-1 levels. For *P. vivax*, the anti-AMA-1 antibody level was significantly (Mann-Whitney U-test: both *P* < 0.05) higher than the anti-SE and anti-MSP1_19_ levels.

Antibody responses were significantly correlated among all antigens, although the strength of correlation varied (Table 2). In general, the correlations were stronger between antibody responses to antigens of the same species (e.g. PfSE *vs* PfMSP-1_19_) than between those of orthologous antigens (e.g. PfMSP-1_19_ *vs* PvMSP-1_19_). Weaker correlations were observed between antibody responses to gSG6 and the parasite antigens (Spearman’s *r* = 0.11–0.20, *P* < 0.01).

**Table 2:** Pairwise correlation (Spearman’s rank correlation coefficient) between IgG responses to parasite and mosquito antigens. All correlations were significant (*P* < 0.01)

		SE		MSP-1_19_		AMA-1		gSG6
Antigen	Species	Pf	Pv	Pf	Pv	Pf	Pv	Ag
SE	Pf							
	Pv	0.5776						
MSP-1_19_	Pf	0.7635	0.4741					
	Pv	0.4171	0.6595	0.4325				
AMA-1	Pf	0.5982	0.4396	0.6074	0.3595			
	Pv	0.4367	0.5821	0.3632	0.465	0.3128		
gSG6	Ag	0.1750	0.1934	0.1168	0.132	0.1937	0.1139	

*Abbreviations*: Pf , *P. falciparum*; Pv, *P. vivax*; Ag, *An. gambiae*

### Seroprevalence in the populations

**Fig. 3:**
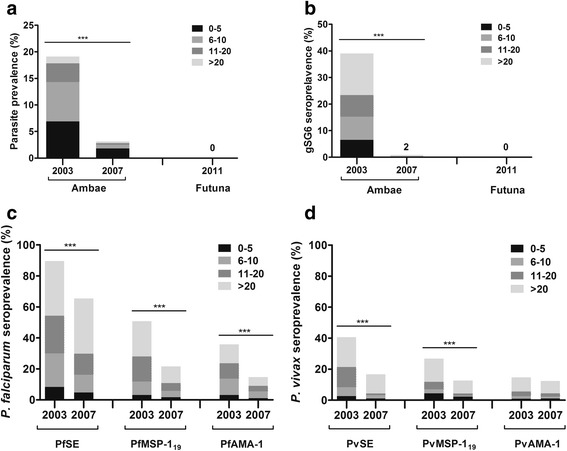
Age-specific parasite prevalence by microscopy and antibody response in Ambae Island and Futuna Island. Graphs are divided by year of sampling and in four age groups for: (**a**) Microscopy, (**b**) *An. gambiae* salivary gland gSG6 antigen, (**c**) *P. falciparum* antigens, and (**d**) *P. vivax* antigens. Serological analyses for *P. falciparum* and *P. vivax* antigens were performed for samples from Ambae Island only. Numbers above bars show the numbers of positive individuals

Figure 3 shows the overall seroprevalence to parasite antigens on Ambae. Among all parasite antigens, seroprevalence to crude SE was highest for both *P. falciparum* and *P. vivax*. Similar to parasite rate and other malaria indices, seroprevalence was significantly higher (Chi-square test or Fisher’s exact test: all *P* < 0.001) in 2003 than in 2007 for all parasite antigens except PvAMA-1. No significant difference was observed in seroprevalence between males and females for any of the antigens tested (Chi-square test or Fisher’s exact test: all *P* > 0.05). For all parasite antigens, the proportion of seropositive individuals significantly increased with age (Chi-square test: all *P* < 0.001). Seroprevalence to gSG6 is shown in Fig. 3b. As expected from an island without anopheline mosquitoes, all samples from Futuna were seronegative. On Ambae, a significant (Fisher’s exact test: *P* < 0.001) decrease in seroprevalence to gSG6 was observed, from 39% in 2003 to 0.7% in 2007. Nevertheless, gSG6 seroprevalence in 2003 was similar among age groups.

### Changes in SCR

**Fig. 4:**
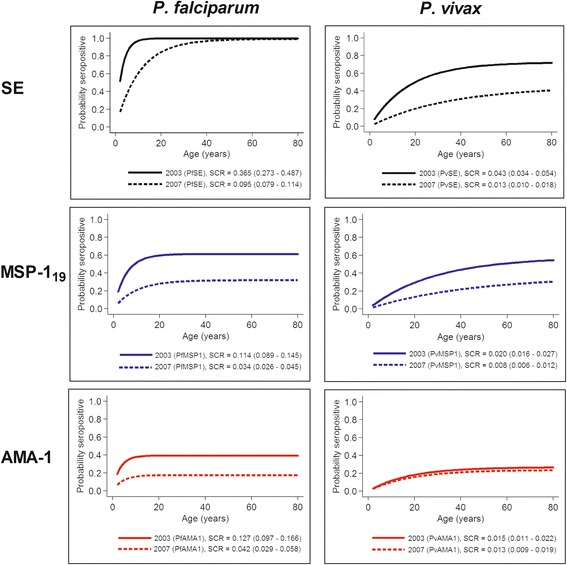
Trends in age-seroprevalence curves and seroconversion rates (SCRs) for all *P. falciparum* and *P. vivax* antigens on Ambae Island. SCR or lambda (λ) was estimated by fitting reversible catalytic conversion models to data from all available age groups. Resulting SCRs and 95% confidence intervals for both years (2003 and 2007) are presented on the graph. The *P. falciparum* fixed seroreversion rates (SRRs) for PfSE, PfMSP-1_19_ and, PfAMA-1 were 0.001 year^-1^ (0.000–0.388), 0.072 year^-1^ (0.042–0.125) and, 0.196 year^-1^ (0.096–0.402), respectively. The *P. vivax* fixed SRRs for PvSE, PvMSP-1_19_ and, PfAMA-1 were 0.016 year^-1^ (0.004–0.063), 0.015 year^-1^ (0.003–0.078) and, 0.042 year^-1^ (0.014–0.131), respectively

The relationship between seroprevalence and age on Ambae was further examined using reversible catalytic conversion models. Seroconversion curves for parasite antigens are shown in Fig. 4. As was observed for the overall seroprevalence, the SCRs for all *P. falciparum* antigens significantly decreased between 2003 and 2007 on Ambae, as evidenced by the non-overlapping confidence intervals. The resulting SCRs suggest at least a three-fold decrease in *P. falciparum* transmission on Ambae. For *P. vivax*, significant decreases in SCRs were observed for SE and MSP-1_19_, but not for AMA-1 between 2003 and 2007. The decreases in SCRs were smaller in magnitude for *P. vivax* antigens than their counterparts for *P. falciparum*.

### Variables associated with seropositivity

We performed univariate and multivariate logistic regression analyses to identify factors associated with seropositivity to any *P. falciparum* and *P. vivax* specific antigens, as well as *Anopheles* gSG6 on Ambae Island (Table 3 **)**. In the adjusted model, the survey year 2007 was significantly associated with lower seropositivity to all antigens tested. Significant associations were found between age and seropositivity to both *P. falciparum* and *P. vivax* antigens. For *P. falciparum* antigens, the likelihood of seropositivity increased significantly with age, whereas for *P. vivax*, the increase in seropositivity was significant among adults aged > 20 only. No association was found between age and seropositivity to the vector salivary gSG6 antigen.

**Table 3:** Logistic regression analyses of seropositivity to *P. falciparum*-, *P. vivax*- and *Anopheles*-specific antigens on Ambae Island. Odd ratios (ORs) and their 95% confidence intervals (95% CI) are presented for both univariate (crude) and multivariate (adjusted) models. Statistical significance was determined using the likelihood ratio test

		*P. falciparum* seropositivity					*P. vivax* seropositivity					*Anopheles* seropositivity				
Variable^a^	Category	*n*/*N* ^b^	Crude OR	*P*	Adjusted OR	*P*	*n*/*N* ^c^	Crude OR	*P*	Adjusted OR	*P*	*n*/*N*	Crude OR	*P*	Adjusted OR	*P*
			(95% CI)		(95% CI)			(95% CI)		(95% CI)			(95% CI)		(95% CI)	
Survey year	2003	143/229	1		1		76/231	1		1		90/231	1		1	
	2007	72/280	0.21 (0.14–0.30)	< 0.001	0.19 (0.12– 0.28)	< 0.001	58/275	0.55 (0.37–0.81)	0.003	0.51 (0.33– 0.77)	0.001	2/282	0.01 (0.00– 0.05)	< 0.001	0.01 (0.00– 0.04)	< 0.001
Age group	0–5	19/102	1		1		20/100	1		1		15/103	1		1	
	6–10	51/109	3.84 (2.06– 7.17)	< 0.001	4.07 (2.09– 7.93)	< 0.001	12/107	0.51 (0.23– 1.10)	0.084	0.47 (0.22– 1.04)	0.062	24/108	1.68 (0.82– 3.41)	0.154	1.58 (0.69– 3.63)	0.280
	11–20	57/111	4.61 (2.48– 8.59)	< 0.001	4.76 (2.45– 9.25)	< 0.001	22/110	1.00 (0.51– 1.97)	1.000	0.93 (0.47–1.84)	0.830	19/111	1.21 (0.58– 2.53)	0.610	0.91 (0.39– 2.09)	0.822
	> 20	88/187	3.89 (2.18– 6.90)	< 0.001	4.30 (2.32– 7.96)	< 0.001	80/189	2.94 (1.66– 5.18)	< 0.001	2.88 (1.62– 5.13)	< 0.001	34/191	1.27 (0.66– 2.46)	0.478	1.15 (0.54– 2.49)	0.714

^a^ Only variables retained in the final multivariate model are listed

^b^ Four samples were excluded (*N* = 509); *n* = number of individuals seropositive to either PfMSP-1_19_ or PfAMA-1, or both

^c^ Seven samples were excluded (*N* = 506); *n* = number of individuals seropositive to either PvMSP-1_19_ or PvAMA-1, or both

## Discussion

Following a gap in funding from external donors that negatively impacted ITN coverage, Vanuatu experienced a resurgence of malaria incidence in the first half of the 2000s. In 2003, Vanuatu secured financial support from the Global Fund to resume distribution of subsidised ITNs. Subsequent decreases in slide positivity rate and proportion of cases caused by *P. falciparum* at the national level were observed after 2006 (16). In this study, we examined the parasite prevalence and IgG antibody responses to antigens from the two major *Plasmodium* species as well as *Anopheles* vector on Ambae Island in 2003 and 2007, marking the peak of the resurgence and its subsidence, respectively. A significant decrease in parasite prevalence was accompanied by significant decreases in seropositivity to most parasite antigens and the vector salivary antigen, suggesting that reinforced vector control played an important role in the reduction of malaria transmission on Ambae.

The shift in species predominance on Ambae between 2003 and 2007 was consistent with the nation-wide decrease in the proportion of *P. falciparum* cases. As ITN was the main intervention tool used during the study period, the shift to *P. falciparum*-*P. vivax* co-dominance in 2007 suggests that ITNs were more protective against *P. falciparum*-infected mosquitoes. Previously in Papua New Guinea, differential ITN protection was partially explained by differences in biting behaviour of *An. punctulatus* mosquitoes infected with different parasite species, with a higher proportion of *P. vivax-*infected mosquitoes biting humans earlier in the evening (25). Furthermore, ITNs provide no protection against *P. vivax* relapse from hypnozoites, which might explain the increase in the proportion of *P. vivax* cases in our study site as well as others, where overall malaria incidences have declined as a result of effective interventions [6, 29, 30].

The greater decrease in *P. falciparum* prevalence between 2003 and 2007 was reflected in the more pronounced reduction in seroprevalence to *P. falciparum* antigens, especially AMA-1 (Fig. 3). The net decrease in seroprevalence to PfAMA-1 (-0.212) was more than nine times higher than the net decrease in seroprevalence to PvAMA-1 (-0.023). In contrast, the net decrease in seroprevalence to crude SE was similar between *P. falciparum* (-0.244) and *P. vivax* (-0.239). It should be noted that crude SE contains multiple proteins, of which some are similar between parasite species [7]. Antibodies to crude SE might therefore not be species-specific, and cross-reactive antibodies might have masked the difference in exposure between *P. falciparum* and *P. vivax*.

Seroprevalence reflects cumulative malaria exposure and can be used to estimate transmission intensity in a population. Modelling changes between seroprevalence and age (i.e. SCR) can help evaluate specific interventions in an area. In practice, SCR is calculated by fitting a reversible catalytic model to the age-specific malaria seroprevalence data, taking into account malaria exposure over time [28]. SCR describes the frequency per unit time (e.g. year) at which seronegative individuals becomes seropositive, and is related to the underlying force of infection [1]. Since serological data integrate exposure over time, they can reveal changes in transmission (e.g. recent outbreak) [3, 7]. In this study, statistical analyses of the seroprevalence profiles suggested that serological response generally increased with age and malaria transmission intensity decreased dramatically on Ambae between 2003 and 2007. SCRs estimated from the age-adjusted seroprevalence curves for *P. falciparum* antigens were higher than the ones for *P. vivax* antigens, reflecting the more intense transmission and the predominance of the former species on Ambae, especially in 2003 (Fig. 3). Changes in *P. falciparum* transmission were readily detected and the estimated decreases in transmission intensity were consistent across the three antigens used: 74.0% for PfSE (SCR from 0.365 to 0.095), 70.2% for PfMSP-1_19_ (0.114 to 0.034), and 66.9% for PfAMA-1 (0.127 to 0.042). For *P. vivax*, the decreases in transmission were more variable: 69.8% for PvSE (0.043 to 0.013), 60.0% for PvMSP-1_19_ (0.020 to 0.008), and 13.3% for PvAMA-1 (0.015 to 0.013). It is unclear why the estimates from PvMSP-1_19_ and PvAMA-1 were drastically different. The difference in SRR, subclass dependent half-life, inherent immunogenicity, and polymorphism or diversity between the two antigens may explain some of the variation in estimates [3]. Similar observations have also been reported previously [7, 31–33].

On Aneityum Island, SCRs decreased by 85.0% (0.04 to 0.006) for PfSE and 93.3% (0.03 to 0.002) for PvSE seven years after the implementation of an integrated malaria elimination programme in 1991 [17]. The observed reductions on Ambae (74.0% for PfSE and 69.8% for PvSE) were less impressive than those on Aneityum, although the study duration on Ambae was shorter (four years). On Aneityum, seroprevalence curves revealed a distinct change in the force of infection corresponding to the abrupt cessation of transmission (19). In contrast, the decrease in transmission on Ambae appeared more gradual (Fig. 3). The absence of a step change in transmission may also be explained by the small size of our samples [34, 35]. Additional follow-up studies with samples from more recent years will be needed to confirm the long-term effect of malaria control interventions on Ambae.

Antibody response to salivary antigen gSG6 was previously shown to be a reliable indicator of human exposure to Afrotropical malaria vectors [19, 20, 36–38]. The SG6 protein first identified in *An. gambiae* [39], was further reported to be highly conserved among a few *Anopheles* species [40] and under purifying selection in an *An. gambiae* population from Burkina Faso [41]. Comparison of SG6 protein sequences among 16 *Anopheles* species [42] showed that the *An. gambiae* gSG6 shares high degrees of identity with orthologues from the main African (*An. arabiensis*, 98%; *An. funestus*, 80%), Asian (*An. stephensi* and *An. maculatus*, 79%; *An. culicifacies*, 72%; *An. sinensis*, 61%; *An. dirus*, 54%) and European (*An. atroparvus*, 66%) malaria vectors, whereas a more limited identity (52%) was found with *An. farauti* (B. Arcà et al. unpublished data). Notably, SG6 is absent in *An. albimanus* and *An. darlingi*, members of the subgenus *Nyssorhynchus* and important malaria vectors in Central and South America. The current study is the first to describe the use of recombinant gSG6 antigen to evaluate exposure to *Anopheles* bites in the Pacific, where all major malaria vectors belong to the *An. punctulatus* group [13]. *Anopheles farauti* (*s.s*.), the only malaria vector in Vanuatu, is absent on Futuna Island (10, 13). Accordingly, all participants from Futuna in this study were seronegative to gSG6 (Fig. 3b). On Ambae, exposure to *Anopheles* mosquito bites was greatly reduced, evidenced by the decrease in seroprevalence to gSG6 from 39% in 2003 to 0.7% in 2007. This reduction in vector exposure suggested the positive impact of ITN distribution after 2003 on vector population density and/or changes in vector behaviour (e.g. preference and aggressiveness towards humans). Nevertheless, a recent survey showed lower ITN use on Ambae (68%) than on Aneityum (73%), where elimination-specific effort has successfully halted malaria transmission [22].

Anti-gSG6 antibody response on Ambae closely followed patterns of decreases in malaria prevalence and community-level antibody responses to all parasite antigens except PvAMA-1. This finding is in agreement with previous reports of malaria incidence and *Anopheles*- and malaria-specific antibody responses in Africa [18, 38, 43]. Previous studies showed the utility of the gSG6 antigen in evaluating the short-term (< one year) efficacy of ITN use [44, 45]. In this study, we demonstrated that gSG6 might also be useful for long-term monitoring. As Vanuatu and neighbouring Solomon Islands embark on malaria elimination (17), serological tools to measure exposure to vectors may become more important as the standard EIR becomes more difficult to determine accurately at low transmission settings (41).

A number of caveats should be considered in this study. The most obvious one concerns the relatively small number of individuals sampled in each year. As small sample sizes might be sufficient to detect a significant reduction in SCR [35], but invariantly lead to poor estimation precision of current SCR and limit the likelihood of identifying significant changes point in malaria transmission over time for the reverse catalytic model [34, 35]. Samples were also collected using a convenience sampling method. Although this approach is valid in obtaining an estimate of antimalarial antibody prevalence [3], it can result in an overestimation of malaria incidence in the area. While the recombinant *An. gambiae* gSG6 antigen could be used to measure exposure to *An. farauti* bites in Vanuatu, the limited conservation between the SG6 protein from these two species (52% identity, 70% similarity) likely result in lower detection sensitivity. Serological analyses using more sensitive biomarker such as cE5 may provide a more accurate measure of exposure to vectors in a population (42). Parasite prevalence in the current study was determined solely by microscopy which likely underestimated the true prevalence. Molecular screening by PCR consistently detects at least twice as many infections as microscopy [46]. Recent studies have confirmed that in malaria-endemic areas sub-microscopic (microscopy negative but PCR positive) *P. falciparum and P. vivax* infections are common [47, 48]. The prevalence of these sub-microscopic infections and their contribution to malaria burden are age-dependent [49]. In areas of very low transmission sub-microscopic carriers are estimated to be the source of 20–50% of all human-to-mosquito transmissions [46]. Considering that malaria transmission has decreased further in Vanuatu, assessing sub-microscopic parasite carriage will be critical in monitoring malaria elimination measures.

## Conclusions

This study showed the decreases in malaria prevalence and antibody responses to crude SE, MSP-1_19_, and AMA-1 from *P. falciparum* and *P. vivax* on Ambae Island, Vanuatu between 2003 and 2007. These decreases were matched by a reduction in seroprevalence to the *Anopheles*-specific salivary gSG6 antigen, suggesting that ITNs were effective in suppressing malaria transmission. This study also demonstrated the utility of recombinant gSG6, originally developed to measure exposure to Afrotropical vectors, in the Pacific where the *An. punctulatus* group is endemic. Larger follow-up surveys are required to examine more subtle changes in transmission.

## Abbreviations

ACT: Artemisinin-based combination therapy
AMA-1: apical membrane antigen 1
EIR: entomological inoculation rate
ELISA: enzyme-linked immunosorbent assay
gSG6: *Anopheles gambiae* salivary gland antigen
ITN: insecticide-treated bed nets
LLIN: long-lasting insecticide treated nets
MDA: mass drug administration
MSP-1_19_: 19 kDa merozoite surface protein-1
OD: optical density
SCR: seroconversion rate
SE: crude schizont extract
